# Unraveling The Link Between Stress and the Severity of Apical Periodontitis: A Systematic Review of Animal Studies

**DOI:** 10.34172/joddd.42826

**Published:** 2026-03-30

**Authors:** Sıla Nur Usta, Antonio Magan-Fernandez, Cangül Keskin, Alper Ceylan, Francisco Mesa

**Affiliations:** ^1^Department of Endodontics, Gulhane Faculty of Dentistry, University of Health Sciences, Ankara, Turkey; ^2^Department of Periodontology, School of Dentistry, University of Granada, Granada, Spain; ^3^Department of Endodontics, Faculty of Dentistry, University of Ondokuz Mayıs, Samsun, Turkey

**Keywords:** Apical periodontitis, Bone loss, Endodontics, Periodontics, Stress

## Abstract

**Introduction:**

Apical periodontitis can occur due to the host immune response, with the release of antibodies, cytokines, and chemokines by stress as a systemic risk factor. This systematic review aimed to investigate the relationship between stress and the severity of apical periodontitis.

**Methods:**

A detailed search strategy was conducted on the "PubMed (MEDLINE), Web of Science (all databases), Scopus, ScienceDirect, EMBASE, and Cochrane Library" databases. Data were screened based on the eligibility criteria. Articles assessing the severity of apical periodontitis using different parameters in stress-induced animals in comparison with stress-free animals were selected. The risk of bias assessment was conducted using SYRCLE’s RoB tool. The GRADEpro tool was used to determine the overall quality of evidence.

**Results:**

Four articles were selected after a full-text assessment. Animals under stress exhibited increased levels of inflammatory cells. The extension of the bone loss and the periapical lesion size differed according to the measurement and visualization methods. All the included articles had a high risk of bias. GRADEpro revealed that the overall certainty of evidence for the included studies was very low.

**Conclusion:**

Under the limitations of this systematic review, an increasing effect of stress on the inflammatory infiltrate was reported in apical periodontitis. Future well-conducted animal studies are warranted to further elaborate on the effects of stress parameters on the severity of apical periodontitis with convenient experimental conditions and advanced evaluation methods.

## Introduction

 The continuation of life depends on homeostasis, defined as maintaining a constant internal environment amidst environmental and external changes.^1^ Stress, as an omnipresent factor threatening homeostasis, is increasingly recognized due to its disruptive effects not only on mental health but also on various physiological processes within the body.^2,3^ Exposure to harmful psychological, environmental, or physiological stressors causes the body to exhibit multifaceted levels of hormonal and immunological responses.^4^ Stressors manifest themselves throughout different life stages, from childhood to elderliness, for several reasons, ranging from socioeconomic dynamics to health concerns and diseases.^5-7^ Stress can be classified as acute and chronic according to the duration and intensity of individuals’ exposure to stressors.^8^

 Apical periodontitis describes periradicular inflammatory diseases characterized by inflammation and destruction of periradicular tissues induced by an infected root canal system or occlusal trauma.^9,10^ Although bacteria and by-products play the main role in infecting the root canal system, the host defense mechanisms have a crucial impact on determining the disease progression.^9^ The occurrence of apical periodontitis involves active processes marked by intricate tissue interactions and is influenced by both the host’s response and the causative agent. In this context, the host releases antibodies, cytokines, and chemokines to mount defense for maintaining the periapical tissue homeostasis.^11^ In asymptomatic forms, apical periodontitis represents a balanced state between the intracanal microbial load and the host. Disruptions to this equilibrium, whether induced by local or systemic disorders, may increase the severity of apical periodontitis.^12^

 A growing number of cross-sectional studies demonstrate an association between apical periodontitis and systemic diseases such as diabetes mellitus and cardiovascular disease.^13‒15^ Additionally, epidemiological studies suggest that psychosocial factors may play a role in the development of apical periodontitis.^16^ In this sense, apical periodontitis can occur due to the host immune response with the release of antibodies, cytokines, and chemokines, with stress as a systemic risk factor.^17,18^ An imbalance created by stress-mediated modulation could contribute to the severity of apical periodontitis by increasing bone loss and inflammatory infiltration. Moreover, the delaying effect of stress on wound healing might also jeopardize the repair capacity of periapical tissues.^19,20^ From an endodontic perspective, elucidating the relationship between stress and disease progression and understanding potential connections is essential to clarify the immunology and pathogenesis of apical periodontitis.

 Several animal studies have demonstrated the additive impact of stress on the severity of apical periodontitis.^18,21-23^ For instance, the effects of stress on inflammatory biomarkers have been shown in rats with marginal periodontitis as an increase in IL-1β and TNF-α levels within peripheral blood and periodontal tissues.^16^ Although IL-1β and TNF-α play a significant role in apical periodontitis as proinflammatory cytokines, some studies have found that stress decreases the levels of local production of IL-1β and TNF-α on wound sites in both human and animal subjects.^24,25^ Conflicting results of studies might stem from the heterogeneity in study designs such as duration of stress exposure, types of stress, and animal models, and indicate that a relationship between stress and apical periodontitis still remains unclear. Therefore, a comprehensive assessment of conducted studies is important to provide a general view regarding this issue by interpreting the quality assessment and level of evidence. To date, no systematic review has examined the methodological quality of studies using animal models linking stress and apical periodontitis. Thus, this study focused on the following question: Is there a correlation between stress and the severity of apical periodontitis? The null hypothesis of the study was that stress would not have an additive effect on the severity of apical periodontitis based on inflammatory infiltrate, periapical lesion size, and bone loss.

## Methods

 This systematic review was performed in accordance with the Preferred Reporting Items for Systematic Review and Meta-Analysis principles (PRISMA) (http://www.prisma-statement.org/).^26^ The protocol, which included detailed criteria for study inclusion and exclusion, search strategy, and data extraction methods, was registered in the PROSPERO database.

###  Database Selection and Search Strategy

 A detailed systematic search strategy was designed by two researchers with experience in endodontics, periodontology, and/or data analysis. A comprehensive search was conducted using “PubMed (MEDLINE), Web of Science (all databases), Scopus, ScienceDirect, EMBASE, and Cochrane Library” databases on 16.02.2024. The determined keywords, including medical subjects heading terms (MeSH) related to the topic, were combined by Boolean operators ‘OR’ and ‘AND’, respectively (Table 1). The language was standardized as English to enhance apprehensibility, and no publication date restriction was applied. Following the application of the search strategy, the received data were exported and compiled in Microsoft Excel 15.0 (Microsoft, Redmond, WA, USA) software to eliminate duplicates and facilitate processability.

**Table 1:** Search strategies and obtained article numbers from databases

	**PubMed**	**Web of Science**	**Scopus**	**ScienceDirect**	**EMBASE**	**Cochrane**
**No.**						
**#1**	(((((((((((((apical periodontitis [MeSH Terms]) OR (periapical periodontitis [MeSH Terms])) OR (alveolar bone loss [MeSH Terms])) OR (alveolar bone losses [MeSH Terms])) OR (chronic apical periodontitis)) OR (apical lesion)) OR (apical infection)) OR (apical inflammation)) OR (periapical lesion)) OR (periapical infection)) OR (periapical inflammation)) OR (endodontic infection)) OR (endodontic inflammation)) **(No. of articles=38,859)**	(((((((((((ALL = (apical periodontitis)) OR ALL = (periapical periodontitis)) OR ALL = (alveolar bone loss*)) OR ALL = (chronic apical periodontitis)) OR ALL = (apical lesion)) OR ALL = (apical infection)) OR ALL = (apical inflammation)) OR ALL = (periapical lesion)) OR ALL = (periapical infection)) OR ALL = (periapical inflammation)) OR ALL = (endodontic infection)) OR ALL = (endodontic inflammation) (**No. of articles=23,468)**	TITLE-ABS-KEY ("apical periodontitis" OR "periapical periodontitis" OR "alveolar bone loss* " OR "chronic apical periodontitis" OR "apical lesion" OR "apical infection" OR "apical inflammation" OR "periapical lesion" OR "periapical infection" OR "periapical inflammation" OR "endodontic infection" OR "endodontic inflammation") (**No. of articles=21,328)**	TITLE-ABS-KEY ("apical periodontitis" OR "periapical periodontitis" OR "alveolar bone loss" OR "chronic apical periodontitis" OR "periapical lesion" OR "periapical inflammation") (**No. of articles=2368)**	ALL ('apical periodontitis' OR 'periapical periodontitis' OR 'alveolar bone loss*' OR 'chronic apical periodontitis' OR 'apical lesion' OR 'apical infection' OR 'apical inflammation' OR 'periapical lesion' OR 'periapical infection' OR 'periapical inflammation' OR 'endodontic infection' OR 'endodontic inflammation') (**No. of articles=12,718)**	TITLE-ABS-KEY ("apical periodontitis" OR "periapical periodontitis" OR "alveolar bone loss* " OR "chronic apical periodontitis" OR "apical lesion" OR "apical infection" OR "apical inflammation" OR "periapical lesion" OR "periapical infection" OR "periapical inflammation" OR "endodontic infection" OR "endodontic inflammation") (**No. of articles=2845)**
**#2**	((stress) OR (chronic stress)) OR (anxiety) **(No. of articles=1,534,215)**	((ALL = (stress)) OR ALL = (chronic stress)) OR ALL = (anxiety) (**No. of articles=2,938,864)**	TITLE-ABS-KEY (stress OR "chronic stress" OR anxiety) (**No. of articles=3,596,274)**	TITLE-ABS-KEY (stress OR "chronic stress") (**No. of articles=682,101)**	ALL (stress OR "chronic stress" OR anxiety) (**No. of articles=2,080,138)**	TITLE-ABS-KEY (stress OR "chronic stress" OR anxiety) (**No. of articles=136,521)**
**Summary**	**#1 AND #2=1418**	**#1 AND #2=1281**	**#1 AND #2=940**	**#1 AND #2=67**	**#1 AND #2=509**	**#1 AND #2=88**

###  Eligibility Criteria

 The study was generated based on the population, intervention, comparison, outcome, and study design (PICOS) strategy as follows:

 Participants (P): Systemic disease-free animals

 Intervention (I): Animals under induced stress protocol

 Comparison (C): Animals without induced stress protocol

 Outcome (O): Inflammatory infiltrate, periapical lesion size, and bone loss.

 Study design (S): Animal studies

###  Inclusion and Exclusion Criteria

 Studies that aimed to evaluate the effect of stress induction on the severity of apical periodontitis based on inflammatory infiltrate, bone loss, and periapical lesion size parameters in comparison with stress-free conditions in animal models were selected. Reviews, case series, letters, pilot studies, in vitro and in vivo studies, finite element analysis studies, conference abstracts, thesis, and animal studies that did not meet the search criteria were excluded.

###  Study Selection

 Two independent researchers screened the titles of articles obtained from duplication-free data. Abstracts of articles were accessed if it could not be decided whether to be included or not solely based on the title review. Moreover, in cases where insufficient information was obtained from the abstracts, a full-text assessment of the studies was performed in terms of eligibility criteria. Where any discrepancies occurred, a third researcher was consulted to reach a conclusive decision, and disagreements between the researchers were discussed until a consensus was reached. After evaluation of the full texts of the relevant articles in detail, those that were deemed to be compatible with the PICOS strategy were included in the study.

###  Data Extraction

 The information obtained as a result of a detailed examination of the included studies in this systematic review by two independent researchers was recorded within the determined parameters. Recorded parameters were as follows: authors, publication year, country of first author, animal and tooth types, sample size, the model of induction of apical periodontitis, induction of stress conditions, groups, evaluation methods, evaluated parameters, results, and main findings. All the researchers approved the final version of the extracted data, followed by the resolution of disagreements.

###  Quality Assessment

 The Systematic Review Centre for Laboratory Animal Experimentation (SYRCLE), which presents a RoB tool for animal intervention studies, SYRCLE’s RoB tool,^27^ was used for the quality assessment of the selected studies. This tool was developed in collaboration with Cochrane to enhance the transparency and applicability of animal studies by assessing the risk of bias more clearly and accurately. ^28^ The RoB tool evaluates six different types of domains as follows: selection bias (sequence generation, baseline characteristics, and allocation concealment), performance bias (random housing and blinding), detection bias (random outcome assessment and blinding), attrition bias (incomplete outcome data), reporting bias (selective outcome reporting), and others (other sources of bias). Each study was classified as having a high risk of bias if it did not meet one or more domains, as uncertain if it partially met one or more domains, and as low risk if it met all the domains. Two researchers independently assessed the above-mentioned domains, and consensus was reached in case of discrepancies.

###  Quality of Evidence

 The level of evidence was assessed using the Grading of Recommendations, Assessment, Development, and Evaluation methodology through the GRADEpro Guideline Development Tool by two independent researchers ^29^ in terms of the following domains: risk of bias, inconsistency, indirectness, imprecision, and other considerations (publication bias, significant effect, plausible confounding, and dose-response gradient). For this systematic review, the risk of bias assessed animal selection, randomization procedures, outcome assessments, blinding, and outcome reporting criteria.^30^ Inconsistency examined the consistency within each study along with other studies, and heterogeneity.^31^ Indirectness evaluated whether the results obtained from the studies could adequately answer the questions focused on within the scope of the study’s hypothesis.^32^ Impression examined the precision of the outcomes and whether any uncertainty in the outcomes was serious enough to downgrade the quality of evidence for that conclusion.^33^ Each domain was deemed as “not serious,” “serious,” and “very serious,” and the overall certainty of the evidence was graded into one of four levels: very low, low, moderate, or high.

## Results

###  Study Selection

**Figure 1:**
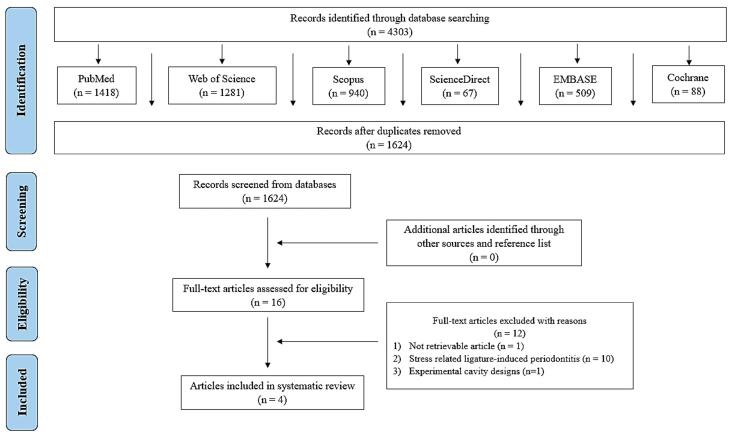
A flow diagram illustrating the systematic review process, including the database searches conducted, the number of abstracts screened, and the full texts retrieved

 A total of 4303 articles were obtained as a result of a detailed electronic search from five databases (Figure 1). Following the removal of duplicates, 1624 articles were screened. Among those, 16 articles that might have been related to the relationship between stress and apical periodontitis were accessed for further evaluation. After a full-text assessment, 12 articles were excluded due to the following reasons: the article was not retrievable, induction of periodontitis via ligature method, and the studies including experimental cavity designs. Consequently, four articles that met PICOS were included in this systematic review.

###  Data Extraction

Table 2 shows the extracted information from selected articles in terms of study characteristics and main findings. Procedures were performed using male Wistar rats in all studies.^18,21-23^ The age of the experimental animals was stated in 3 articles as 60 days and 90 days old.^18,22,23^ Mandibular molars were accessed in all studies,^21-23^ except one study in which maxillary molars were used.^18^

**Table 2:** Summary of the main characteristics and results of the included studies

**Author, year, and country**	**Animal characteristics**	**Sample size**	**Tooth** **type**	**Induction method of apical periodontitis**	**Induction of stress**	**Groups**	**Evaluation methods**	**Evaluation criteria**	**Results and ** **main findings**
Gomes et al.^23^ 2019 Brazil	60-day-old Wistar male rats 280–350 g	25 animals	Mandibular molars	- Pulp exposures were performed using diamond tips. - The teeth were conditioned for pulp exposure to induce bacterial contamination and the consequent development of inflammatory apical periodontitis lesions.	- Conditioning fear stress sessions for 50 consecutive days in a conditioning fear stress chamber	-Stress-induced animals -Control group	Radiographic and histologic analysis	**-**Intensity of radiopacity -Bone loss -Thickening of the PDL -Inflammatory response	- Stressed animals presented increased levels of bone loss and inflammatory cells in the root apex in comparison with the control group. - No radiographic differences were observed between the groups.
Khoury et al.^21^ 2020 Brazil	Male Wistar rats 180 g	32 animals	Mandibular molars	- Pulp exposures were performed using a steel bur. -The pulp chamber remained exposed for 32 days to the oral cavity for periapical lesion induction.	-Two periods of food or water deprivation (overnight) -Two periods of 45° cage tilt (overnight) -Two periods of cage soiling (overnight) -Two periods of stroboscopic light -Two periods of 4°C or 45 °C swimming -Two periods of tail clamping -Two periods of no stress.	-Non-stressed (NS) -Stress + saline solution (SS) -Stress + β-adrenergic blocker propranolol (Sβ) -Stress + α- adrenergic blocker phentolamine (Sα)	Radiographic and histologic analysis	-Tissue, bone, and periapical lesion volumes -Corticosterone levels -Inflammatory response -Immunohistochemical response	-The SS group displayed significantly higher corticosterone levels than the NS group. -Higher IL-1β serum level was observed in the NS group compared to all stressed groups. -All groups presented similar periapical lesions with moderate inflammatory infiltrate. - All groups presented moderate inflammatory infiltrate, without statistically significant differences between them. -Rank-L/OPG system and periapical lesion volumes were not affected.
Minhoto et al.^22^ 2021 Brazil	90-days-old male Wistar rats ~370 g	58 animals	Mandibular molars	- Pulp exposures were performed using a carbide bur. - The pulp tissue was disorganized with a size of 10 K-file and remained open to the oral cavity to induce periapical lesions.	- Food or water deprivation (overnight) - Cage soiling (overnight) - 45° cage tilt (overnight) - Swimming at 4 °C or 45 °C - Exposure to strobe light (2 h) -Two periods of no stress.	-Animals with apical periodontitis (AP) - Animals with apical periodontitis + stress (AP + S)	Radiographic and histologic analysis	-Serum corticosterone levels -Tissue, bone, and periapical lesion volumes -Inflammatory response	- The AP + S group had a significantly lower average percentage of weight gain. - Significantly higher levels of corticosterone were found in the AP + S. - The AP + S group had a significantly greater intensity and extension of inflammatory infiltrate with larger areas of bone loss compared to the AP groups. - The volume of the periapical lesions in the AP + S group was significantly larger than that of the AP.
Botelho et al.^18^ 2023 Brazil	90 days male Wistar rats	40 animals	Maxillary molars	- Pulp exposures were performed using a surgical round burr. -The cavities were standardized with a diameter of 0.5 mm and remained exposed to the oral environment for 40 days, allowing the periapical lesion formation.	- Early life stress was induced by maternal separation. - Rats were separated from their mothers for 3 h a day and transferred to another box, containing a thermal blanket with regulated temperature.	-Animals without stress and apical periodontitis (control) -Animals with stress (S) -Animals with apical periodontitis (AP) -Animals with stress and apical periodontitis (AP + S)	Histological and histometric analysis	-Periapical inflammation and bone resorption -Inflammatory response -Anxiety levels	-The intensity of the inflammatory infiltrate was significantly larger in the AP + S group when compared with AP group. -The AP + S group exhibited significantly greater alveolar bone loss, with a periapical lesion size compared with the AP group. -Rats with AP displayed higher anxiety-like behavior in relation to the control group. -Exposure to early life stress abolished the AP-induced increased anxiety-like ‘behaviour.

 In terms of induction of apical periodontitis, pulp exposures were performed mechanically by different types of burrs, and pulps were left open to oral contamination at various time periods. Furthermore, physiological stress was induced in animals using varied indicators, such as conditioned fear,^23^ food or water deprivation,^21,22^ cage tilt,^21,22^ cage soiling,^21,22^ stroboscopic light exposure,^21,22^ swimming,^21,22^ tail clamping,^21^ and maternal separation.^18^

###  Main Results

 Gomes et al.^23^ demonstrated that although stress-induced animals had increased levels of bone loss and inflammatory cells in the root apex in comparison with the control group, no radiographic differences were observed between the groups. Khoury et al.^21^ observed the higher corticosterone levels and inflammatory cytokines in a stress-induced group. However, this study reported no comparable changes between groups regarding the Rank-L/OPG system and periapical lesion volumes. In the study performed by Minhoto et al.^22^ higher levels of corticosterone and greater intensity and extension of inflammatory infiltrate with larger areas of bone loss were presented. In addition, the volume of the periapical lesions in the stress-induced group was significantly larger than the control group. Finally, Botelho et al.^18^ indicated that the stress-induced group had a larger intensity of the inflammatory infiltrate and exhibited greater alveolar bone loss with a periapical lesion size. Additionally, stress-induced animals displayed higher anxiety-like behavior.

###  Quality Assessment

**Figure 2:**
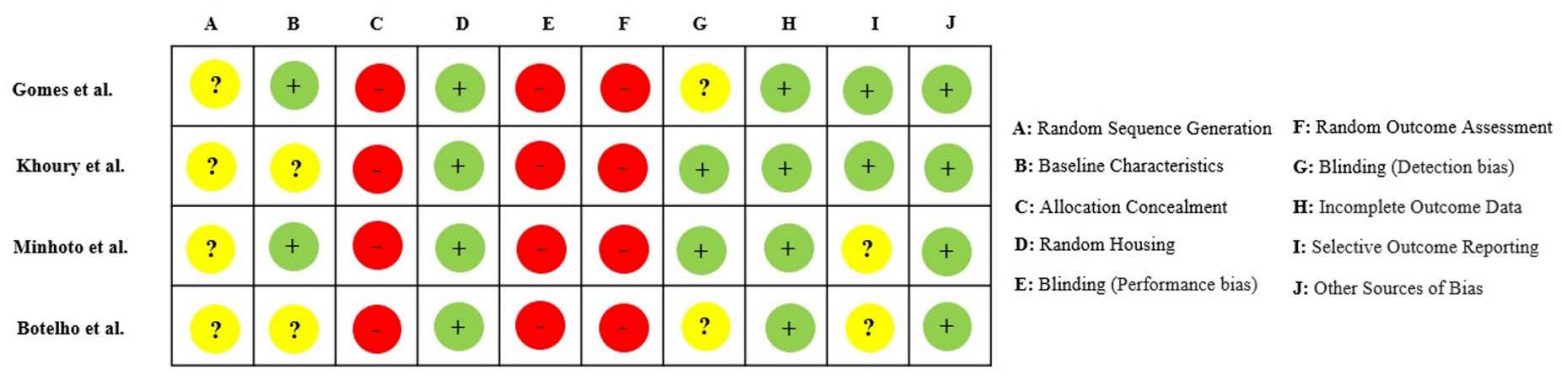
Summary of the assessment of the risk of bias for each selected article

Figure 2 demonstrates the risk of bias assessment. Based on the evaluation, all the selected articles were considered as having a high risk of bias. Accordingly, domains of allocation concealment, blinding, and random outcome assessment were categorized as high risk for all the included studies. Although random sequence generation was performed, the details were unclear.^18,21-23^ Furthermore, apart from two studies,^22,23^ some baseline characteristics of the used animals were missing; therefore, these studies were classified as having unclear risk of bias.^18,21^ Since the housing conditions were well-described, the random housing domain was considered as low risk of bias for all selected studies. While two studies indicated the presence of blind outcome assessors from knowing which intervention each animal received,^21,22^ this domain was unclear for the other two.^18,23^ Incomplete outcome data were unclear for two studies since there was a lack of information regarding some parameters.^22,23^ Finally, selective outcome reporting domains presented a low risk of bias for all the studies.

###  Quality of Evidence

Table 3 presentsthe results of the evaluation of the quality of evidence using GRADEpro. The certainty of the evidence was found to be overall of “very low” quality of selected studies that received “very serious” risk of bias and “not serious” for indirectness. Since all the results were not totally consistent across studies, the inconsistency domain was deemed as “serious.” Furthermore, the imprecision domain was also considered as “serious” due to the uncertain results reported in studies.^18,22^ Moreover, any considerations such as large effect, plausible confounding, and dose‒response gradient were not identified. Additionally, no data verification could be performed that would upgrade the certainty of the evidence.

**Table 3:** Assessment of quality of evidence using GRADEpro

**Certainty Assessment**						
**No of studies**	**Risk of bias**	**Inconsistency**	**Indirectness**	**Imprecision**	**Other considerations**	**Certainty of evidence**
4	Very serious ^a^	Serious ^b^	Not serious ^c^	Serious ^d^	None	Very low

GRADEpro: Grading quality of evidence and strength of recommendations. ^a^All the selected studies showed very serious limitations that downgraded the quality of the evidence. ^b^The results were not totally consistent across studies. Khoury et al. used similar methods to other studies but reported different results. ^c^The evidence directly answered the questions that have been investigated. ^d^The results of the studies that have been performed by Minhoto et al. and Botelho et al. had some deficiencies and contradictions in terms.

## Discussion

 The physiological effects of various stress factors on chronic inflammatory diseases such as apical periodontitis have been demonstrated in the literature.^18,34,35^ Psychological stress can indirectly modulate the repair process by promoting the adoption of health-damaging behaviors with increased levels of glucocorticoids and proinflammatory cytokines.^25,36^ Examining the effects of stress on the release of inflammatory mediators, bone loss and periapical lesion size associated with apical periodontitis may lead to a better identification of the causative factors of the disease and versatile application of its treatment. In this sense, systematic reviews are comprehensive and unbiased studies that evaluate related articles’ synthesis of evidence on a clearly presented topic using critical methods.^37^ Since the application of stress factors on humans poses ethical concerns, studies are carried out in a more favorable and controllable manner through animal experiments. Thus, this systematic review of animal studies aimed to unravel the link between stress and apical periodontitis, comparing related parameters of the severity of the disease. Since stress was found as a contributing factor for the severity of apical periodontitis on bone and surrounding tissue, as shown in the majority of the studies, the null hypothesis was rejected.

 The physiopathological aspects of apical periodontitis have been studied in many instances in rats since rats possess several qualities such as smaller sizes, known genetic background, and similarities to human disease conditions that make them a highly suitable and favored animal model.^38,39^ In this sense, experiments were conducted using rat models in all of the selected studies in this systematic review. Although rat models allow the evaluation of many different aspects of apical periodontitis regarding gene expression, inflammatory regulatory mechanisms, resolution, and healing processes, differences in the extent of development, activation, and aggression of the disease process between rats and humans should be considered.^40,41^

 The induction method of apical periodontitis is a crucial factor that affects the course and degree of the disease. In animal models, periodontitis is commonly induced by three accepted methods, including placement of a ligature around the maxillary or mandibular teeth, oral bacterial inoculation, and lipopolysaccharide (LPS) injection.^40^ Although these methods have various advantages and disadvantages compared to each other, they have been successfully applied for the induction of apical periodontitis in animals in the literature. In all the included articles in this systematic review, the induction of apical periodontitis was performed by pulp exposure to oral flora that leads to bacterial contamination and the consequent development of inflammatory apical periodontitis lesions. Using the same induction model in all selected studies may warrant the development process of apical periodontitis to be similar, and consequently allow a more accurate and effective comparison of the baseline data.

 Animal models of stress-related disorders evaluate the effect of stress on several diseases by inducing the occurrence of human psychiatric disorders in animals using various methods.^42^ The main differences between these methods are the age, gender, duration (chronic or acute), and nature of the stressor.^42^ In this systematic review, two of the four articles induced stress by applying food or water deprivation, cage tilt, cage soiling, stroboscopic light, tail clamping, and swimming.^21,22^ Gomes at al.^23^ created a stress environment by conditioning fear-stress sessions in a conditioning fear-stress chamber.^23^ Moreover, Botelho et al.^18^ used the early life stress method induced by maternal separation. Different stress stimulants may cause an increase in cortisol levels at various rates, consequently affecting the progression of apical periodontitis.^43^

 Based on the present findings of the included articles in this systematic review, three out of four reported increased levels of inflammatory infiltrate in stress groups.^18,22,23^ However, interestingly, Khoury et al.,^21^ who analyzed the inflammation process by examining interleukins (IL-1β, IL-6, IL-10, and IL-17) macrophages, lymphocytes, neutrophils, along with corticosterone, did not find a statistically significant difference in terms of inflammatory infiltrate apart from the corticosterone level. The authors explained this finding by any differences that can only be detected at a cellular or molecular level. On the other hand, Minhoto et al.^22^ did not indicate the type of inflammatory cells. While Gomes et al.^23^ assessed the leukocytes and mast cells along with corticosterone, Botelho et al.^18^ evaluated macrophages and lymphocytes. Although the different cell types examined in the included studies are involved in inflammatory events, a direct comparison cannot be made in this study because each cell is related to the processes associated with a different marker.

 Regarding bone loss and periapical lesion size, the included studies presented contradictory results. Gomes et al.^23^ showed an increased bone loss area in histopathological analysis; however, this difference could not be found in the radiographic evaluation. Moreover, in the study of Minhoto et al.,^22^ periapical lesion volume and bone loss area that were measured by micro-computed tomography (micro-CT) and histological methods, were significantly larger related to stress. This difference can be attributed to the inadequacy of two-dimensional radiographs in showing periapical changes compared to three-dimensional techniques.^44^ Furthermore, Botelho et al.^18^ measured bone resorption and periapical areas by histomorphometric analysis and revealed significantly greater alveolar bone loss with a periapical lesion size related to stress. Khoury et al.^21^ evaluated the bone volume and periapical lesion volumes with micro-CT in addition to the histomorphometric analysis of the periapical lesion area and revealed no significant difference. Type of stress, duration, and evaluation methods may be the factors affecting the outcomes in stress-induced groups.

 Using systematic reviews to obtain evidence-based information in healthcare is common in clinical practice.^27^ However, the extent to which systematic reviews can produce reliable results depends on the quality of the included studies and the risk of bias.^45^ In this systematic review, all the included studies were considered as having a high risk of bias. In particular, deficiencies in allocation concealment, being blinded to applied interventions, and random outcome assessment domains had presented higher risks. Moreover, a random component in the sequence generation process in the selected studies was not clear. Gomes et al.^23^ and Botelho et al.^18^ did not provide any information concerning blinded outcome assessment. Additionally, while Minhoto et al. ^22^ have not indicated the inflammatory cell types, there was a contradiction in terms of bone resorption and periapical lesion size in the study of Botelho et al.^18^

 Assessment of the certainty of evidence ratings in animal studies is another essential process to strengthen health recommendations; therefore, it needs to be inspected using certain guidelines. These guidelines also contribute to the overall improvement of research practices and the ethical treatment of animals in scientific investigations. In this systematic review, the GRADEpro tool was used to check the certainty of evidence. Based on the findings, the studies were deemed to have a ‘very serious’ risk of bias evidence since they showed very serious limitations that downgraded the quality of the evidence. Although Khoury et al.^21^ used similar and good-quality tools for evaluation, their results were not consistent across other studies. Furthermore, the results of Minhoto et al.^22^ and Botelho et al.^18^ were not precise enough to demonstrate each parameter in detail. Thus, the overall certainty of evidence for the included studies was low.

 The limitations of this systematic review include differences in stress induction methods and heterogeneity in assessment methods of included parameters along with high-risk bias, and downgraded quality of evidence ratings. Therefore, the results of the conducted studies should be evaluated carefully. In addition, the absence of standardized outcome evaluation and varying follow-up times in the included studies challenges direct comparison between their findings. However, since hormonal balances have a crucial effect on the process of apical periodontitis, it is important to evaluate various stress models by conducting animal experiments. This systematic review warrants that well-designed and detailed studies with a wide range of assessment scales are needed to evaluate the effect of stress on the severity of apical periodontitis.

## Conclusion

 Under the limitations of this systematic review with a high risk of bias and very low certainty evidence, an increasing effect of stress on the inflammatory infiltrate was reported in apical periodontitis. The extension of the bone loss and the periapical lesion size differed according to the measurement and visualization methods. Corticosterone levels were significantly higher in stress-induced groups.

## Competing Interests

 The authors deny any conflicts of interest.

## Ethical Approval

 None.
